# A Sweet Potion to Put Embryonic Stem Cells to Sleep

**DOI:** 10.1100/tsw.2009.25

**Published:** 2009-03-31

**Authors:** Kaushik D. Deb

**Affiliations:** Embryonic Stem Cells Program, Manipal Institute of Regenerative Medicine, #10 Service Road, Domlur Layout, Bangalore 560071, India

**Keywords:** trehalose, cryopreservation, off the shelf, toxicity, epigenetics, storage, transportation, embryonic stem cells, embryos, natural cryoprotectants, biopreservatives, banking, therapy, drug screening

## Abstract

Human embryonic stem cells (hESCs) are rapidly revolutionizing the areas of drug screening and therapy. In view of their applications and high operational costs at global multicentric setups, the ability to store and transport hESCs and derivatives under ambient temperatures, and their cryopreservation without compromising the stemness, function, and viability, is becoming imperative. Here we discuss the need for a natural cryoprotectant and biopreservative with a potential to improve cryopreservation, ambient temperature storage, and shipping of hESCs and derivatives. Trehalose, a naturally occurring disaccharide with therapeutic properties, protects the integrity of cells against desiccation, dehydration, and extreme heat or cold, and has been successfully tested for some somatic stem cell types. However, the biggest setback is the inability of mammalian cells to internalize trehalose. Here we review the methods being developed at different laboratories to facilitate its intercellular transport and advocate the need for similar advances in hESCs.

## INTRODUCTION

In the last few decades, advances in embryonic stem cell research have provided unprecedented opportunities for studying mammalian developmental programming, lineage induction, and differentiation to tissues for therapy and drug development[1,2,3]. Human embryonic stem cells (hESCs) are being differentiated to tissue progenitors of different lineages, and are being successfully used in drug screening platforms and therapies, some of which are now close to approval. Therapeutic applications of hESCs for various diseases in animal models are already showing encouraging results[2]. However, therapeutic applications of differentiated hESCs carry a risk of teratoma formation by contaminating undifferentiated hESCs and are therefore restricted[2]. Further, recent progress on selective ablation of undifferentiated hESCs after their differentiation to tissues may actually lead to a faster translation of hESCs from lab to bedside than is envisioned[4]. Central to these issues are questions, such as: How are the hESCs derived and how are they cryopreserved? Were the embryos used for their derivation preserved with toxic cryoprotective agents, etc.? All these facts would also have implications in drug screening and are of serious concern for the translation of hESCs from bench to bedside.

With advances in drug screening and cell replacement therapies, a need to transport hESCs and progenitors across long distances is arising, preferably at ambient temperatures, while maintaining cell viability and function. Such advances are required in order to address medical emergencies where immediate stem cell transplantations are vital. Conventional storage and shipping of cells in liquid nitrogen not only reduces the efficacy of the cells (following freeze-thaw cycles), but is also time consuming, expensive, and often cumbersome. In order to use hESCs in therapeutic research or drug discovery, satisfactory cryopreservation technologies are crucial for the establishment of hESC banks, which preserves disease lines or genetically modified hESC clones[5,6]. Several reports suggest that the characteristics of hESCs tend to change during long-term culture, storage, and passaging[7,8].

With rapid globalization of the stem cell business and research, it is becoming increasingly important to be able to transport cells from the lab to other centers with induction of minimal genetic and epigenetic changes and with cost effectiveness. Shipping cells by air in liquid nitrogen is also becoming a safety issue for many carrier airlines. To address such requirements, multicentric trials towards the shipping of various types of stem cells and their differentiated derivatives at ambient temperatures across laboratories or medical centers using natural preservatives are currently ongoing at our own research centers and at different laboratories throughout the world[9]. Somatic cells are being transported by road and by air, and the different methods have met with varying degrees of success. Once established, such methods would have tremendous practical and economical advantages over conventional liquid nitrogen storage and shipping.

Several pharmaceutical companies are using hESCs as models for *in vitro* research in drug discovery and toxicology[10]. Difficulties, such as the lack of standard protocols in the establishment and storage of the different hESC lines, are annoying obstacles in the interpretation of different hESC line behaviors in the laboratory. The creation of stem cell banks could help in the development of standardized protocols of hESC culture and to monitor epigenetic changes caused by culture pressure, cryoprotectants used, and the preservation conditions[11]. Two methods widely used to cryopreserve hESC lines before preservation or transportation are slow freezing–rapid thawing[12,13,14] and vitrification[15,16]. However, these techniques have several drawbacks as discussed in the following sections of this review. We will also look at the adverse effects exerted by the cryoprotectants, and revisit the needs for safer and natural cryoprotectants.

To make stem cell–based transplantations and drug screening programs economical, and to have a consistency in their nature, behavior, and performance, it is necessary to (1) develop ways to arrest cells in an unaltered state at ambient and/or freezing temperatures and (2) to maximize their recovery with minimum injuries and genomic and epigenomic alterations induced by the procedures. The biotechnology and pharmaceutical industry is now searching for molecules that can stabilize the cellular protein and membrane at adverse environmental conditions like dehydration, extreme low and high temperatures, and oxidation. Trehalose is one such molecule that has attracted a lot of attention for this purpose and for studying several neurodegenerative diseases that are associated with the misfolding of disease-specific proteins[17,18]. This naturally occurring disaccharide is widespread throughout the biological world in over 80 species representing plants, algae, fungi, yeasts, bacteria, insects, and other invertebrates[19]. In this review, we discuss the unique properties of trehalose and evaluate the advantages of using trehalose for short-term cell storage at room temperatures or long off-the-shelf cell storage, and in using it as a cryoprotectant during long-term preservation of the hESCs. We also review the methods that are being developed at laboratories across the world to facilitate its intercellular transport in somatic and adult stem cells, and demonstrate the need for similar advances in hESCs.

## CELL STORAGE: CRYOPROTECTIVE AGENTS AND NATURAL BIOPRESERVATIVES

Storage of cells usually means the maintenance of cells in a biologically inactive state without loss of function and viability. Conventionally, such storage is done by cryopreservation in liquid nitrogen using cryoprotective agents (CPAs) to protect the cells from cryoinjuries and other changes. Once stored, the growth-arrested cells can be transported in liquid nitrogen tanks/cryoshippers from one place to another. However, such storages have many disadvantages related to the logistics of cross-continental shipping and recovery of cells. We and several other groups around the world, therefore, are trying to develop methods for long-term cell storage at room or ambient temperatures using naturally occurring cell/biopreservatives. Many of the naturally known cryoprotectants are also known to offer protection to cells during extreme temperatures and dry states, and thus offer possible applications in both cryopreservation and dry desiccated storage at ambient temperatures. This section presents a brief overview of the various types of widely used CPAs.

Cryopreservation protocols typically require the use of CPAs, either synthetic or natural, to maximize the clinically relevant survival rates of cells. A variety of molecules have been tried; however, the two most commonly used substances are glycerol and DMSO (dimethyl sulfoxide). Other substances used include sugars, polymers, alcohols, and proteins. CPAs can be divided roughly into two different categories: (1) permeating CPAs: substances that permeate the cell membrane; and (2) nonpermeating: impermeable substances. The addition of a CPA is known to change the ionicity of a solution. Both tissues and intact organs can show a decline in cellular viability when exposed to sufficiently large step changes in external osmolarity caused by the presence of a freezing solution[20]. In addition, long-term exposure to low concentrations of certain CPAs at room temperature is potentially damaging to the cell membrane and toxic to the cells[21]. Organic CPAs (e.g., DMSO and glycerol) have a colligative effect on water properties, but also affect cellular structures (e.g., the membrane, actin, or tubulin cytoskeleton). In addition, they are highly complex molecules and may add a number of factors (known and unknown) that can interfere with or alter cellular function[22]. Other nonpermeating, natural, and protective agents (e.g., sucrose and trehalose) have been studied for their effectiveness as cryoprotective agents for cells with variable results. However, on internalization, a natural cryoprotectant like trehalose can be more efficient than the conventionally used CPAs. The following sections of this review attempt to explain why trehalose could be a better CPA than other synthetic and naturally available CPAs.

## PROPERTIES AND FUNCTIONS OF TREHALOSE

Trehalose, a naturally occurring cryoprotectant, was first discovered by Wiggers in 1832 in an ergot of rye and in 1859, Berthelot isolated it from trehalamanna (manna) made by weevils, and named it as trehalose. Trehalose, also known as mycose, is a disaccharide in which two glucose molecules are linked together in α, α-1, 1-glycosidic linkage (Fig. 1). It is a nonreducing sugar that is not easily hydrolyzed by acid and the glycosidic bond is not cleaved by glucosidase[23]. Trehalose is a source of energy in many bacteria, fungi, insects, plants, and invertebrates, and is implicated in anhydrobiosis, which is the ability of plants and animals to withstand prolonged periods of desiccation[23,24,25]. For example, a chironomid, *Polypedilum vanderplanki,* is the largest multicellular animal known to tolerate almost complete dehydration without damage. The ability of *P. vanderplanki* larvae to accumulate a large amount of trehalose rapidly (18% of dry body mass) enables it to adapt to the adverse conditions[26]. Cryptobiotic larvae show extremely high thermal tolerance from –270 to +106°C and can recover soon after prolonged dehydration of up to 17 years[27]. Because of its high water-retention capabilities, trehalose is used in food and cosmetics. Whereas some of the protective effects exerted by trehalose may be explained by its chemical chaperone properties, its actions largely remain unknown. Trehalose has been shown to have good stabilizing functions, namely, preventing starch retrogradation, protein denaturation, and lipid degradation. It stabilizes bioactive soluble proteins, such as monoclonal antibodies and enzymes for medical use[28,29].

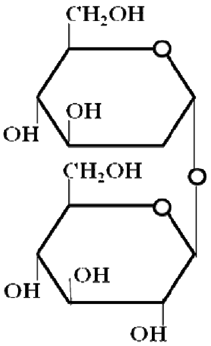

**Trehalose**
**($C_{12}H_{22}O_{11}$)**

**FIGURE 1.** Molecular structure of trehalose.

Two main hypotheses have been proposed to explain the protective effect exerted by trehalose: (1) the water-replacement hypothesis and (2) the vitrification hypothesis. The water-replacement hypothesis proposes that trehalose can take the place of water molecules and form direct hydrogen bonds with the polar or charged groups of biomolecules, thereby stabilizing the native structure of proteins or phospholipid membranes in the absence of water. Trehalose is thought to act by altering or replacing the water shell that surrounds lipid and protein macromolecules[29]. It is thought that its flexible glycosidic bond allows trehalose to conform to the irregular polar groups of macromolecules. Thus, it is able to maintain the three-dimensional structure of these biologic molecules, preserving biologic function. Studies in multiple biologic systems established its ability to inhibit lipid and protein misfolding[30]. Trehalose, therefore, became an attractive molecule for study in neurodegenerative diseases like Huntington's disease, which is characterized by protein misfolding and aggregate pathology[17,18]. Although trehalose absorption in human cells has not been well studied, a small fraction (0.5%) is likely to be absorbed by passive diffusion or via a fluid-phase endocytotic mechanism, as has been demonstrated for other disaccharides[31,32]. In mammalian cell culture, once introduced, intracellular trehalose concentration does not change for extended periods of time (from days to weeks)[33]. The second hypothesis, the vitrification hypothesis, proposes that sugar molecules protect the biomolecular/bilayer membrane structures by forming amorphous glasses, which would reduce structural fluctuations and prevent denaturation or mechanical disruption. However, these mechanisms are not necessarily exclusive to each other. Besides application as an excellent cryoprotectant, this property of trehalose could provide an advantage for designing techniques for dry desiccated and ambient temperature storage of mammalian cells.

## TREHALOSE VS. OTHER NATURAL SUGARS AS CRYOPROTECTANTS

The cell membrane (plasmalemma) is the site of most freezing damage. Therefore, good cryoprotectants may not only perform the antifreeze function of preventing ice formation, but may also protect cell membranes, organelles, or macromolecules, with proteins being the most vulnerable. The two disaccharides that most protect proteins and cell membranes against chilling, freezing, and dehydration are sucrose (fructose, glucose) and trehalose (glucose, glucose). Sucrose is the most common sugar found in freeze-tolerant plants. Both sugars fit well in cell membranes, binding to phospholipid head groups. Both trehalose and sucrose are nonreducing sugars because their glycosidic bonds prevent opening of hemiacetal bonds. In acidic conditions, however, sucrose is far more vulnerable to hydrolysis into its reducing-sugar monosaccharides than trehalose[34]. This indicates that trehalose may be a better choice over sucrose in such applications.

Trehalose also has a hydration radius that is 2.5 times greater than that of sucrose, and 2.5 times the concentration of sucrose is required to provide an equivalent amount of protein protection[35]. Trehalose protects cells and proteins from oxidative damage, whereas sucrose does not[36]. Trehalose interacts more strongly with water than does sucrose, at least partly because sucrose forms intramolecular hydrogen bonds[37]. At the phospholipid bilayer of cell membranes, trehalose is able to displace water molecules bound to carbonyls, but sucrose is not[38].

When used for vitrification, both sucrose and trehalose form glass in the dry state. By definition, a glass is a liquid of such high viscosity that it slows chemical reactions or even stops them altogether. Such a stage or glass formation (vitrification) is often required to stabilize dry biomolecules. This viscous glass can, however, be readily melted by adding water, thus restoring conditions that allow normal metabolism in cells. This is very important when considering the stability of cells in such a dry state. Polar head groups of phospholipids are normally hydrated to some extent and are separated from each other by these water molecules. When these phospholipids are dehydrated, the packing density of the head groups increases, thereby increasing van der Waals interactions among the hydrocarbon chains. As a result, the phase transition temperature ($T_m$, the temperature at which the hydrocarbon chains undergo a change from gel to liquid crystalline state) increases enormously. This would mean that dehydrated lipids enter the gel phase at temperatures at which hydrated lipids are in the liquid crystalline phase[41]. Vitrification of the sugar inhibits the increase in $T_m$ during dehydration by limiting lateral stresses in bilayers during the process. Trehalose has a higher glass transition temperature ($T_g$) than sucrose. At 5% water content, $T_g$ for trehalose is found to be 115ºC, whereas $T_g$ for sucrose is about 65ºC[39]. As a result, the addition of a small quantity of water to sucrose or by absorption of moisture from the air decreases $T_g$ to below the storage temperature, whereas at an equal amount of water content, $T_g$ for trehalose remains above the storage temperature. Under such conditions, the sample dried with sucrose would degrade more rapidly than that in trehalose.

Trehalose and sucrose both have been reported to be efficient cryoprotectants, which is ascribed to the replacement of water at the membrane structure[40]. Trehalose is effective in replacing water when the bilayers were dried under drastic conditions[40,41,42,43]. However, it is not clear how many water molecules can be displaced by trehalose and sucrose from the lipid bilayer in excess of water. As the structure and dynamics of the membrane are influenced by the surrounding medium and, vice versa, the membrane (lipids) exerts a strong influence on the liquid vicinity[44], it is important to know how many of those water molecules affect the magnitude of the dipole potential, which can be replaced by sugars in excess of water, and which are the sites or chemical groups at which the exchange with water is taking place. Knowledge of these details would give insight into the mechanisms of the protective action of the different saccharides for membranes under stress. Fourier transform infrared spectroscopy (FTIR) study under the same conditions indicated that trehalose binds to the carbonyl groups, while sucrose showed no specific binding[38]. It was concluded that per lipid molecule, 11 of 14 water molecules can be replaced by three trehalose molecules. About four are displaced by changes in the water activity of the bulk solution and seven by specific interactions with the phospholipids. In this last case, at least two of them are linked to the carbonyls and this appears to be the cause of the decrease in the dipole potential of the membrane. In contrast, four sucrose molecules displace only three water molecules per lipid, with no effect on the dipole potential or the carbonyl groups[38]. With such novel properties, it seems obvious that trehalose is superior to other sugars in membrane protection, stabilization, and cryoprotection during extreme dry/cold conditions.

## ADVANTAGES OF TREHALOSE OVER CONVENTIONAL CPAs IN STEM CELL BANKING AND THERAPY

### Therapeutic Aspects

Several reports on the traditional methods of using DMSO as a CPA for stem cell preservation before transplantation have shown adverse effects on the patients following therapy[45]. The level of toxicity experienced by the patients is related to the amount of cells cryopreserved with DMSO[46]. Infusion of DMSO-preserved peripheral blood progenitor cells (PBPC) in patients is often associated with toxic reactions, such as nausea, vomiting, cardiac dysfunction, anaphylaxia, acute renal failure, hypotension, and transient hypertension. In addition to the multiple toxic effects of DMSO, the number of dead and injured cells infused during transplantation may also cause side effects, such as fever and abdominal cramps[47,48]. Apparently, it has become necessary to reduce the concentration or eliminate the use of DMSO in cryopreservation of stem cells for therapies and use natural CPAs, such as sucrose, trehalose, or other sugars.

### Antiapoptotic Activity

Most existing methods for banking do not facilitate sufficient recovery of viable cells to meet the needs of basic research or clinical trials and transplantation[49]. Apoptosis associated with cryopreservation failure has been identified as a strong cause for cryopreservation-associated cell death in hESCs[50]. Baust et al.[51] reported up-regulation of transcriptional and proteolytic activity of caspase-3 following "classical" cryopreservation using DMSO. They also reported a transcriptional up-regulation of caspase-8 and -9 following thawing, leading to caspase-3 activation and cellular demise. Stroh et al.[52] demonstrated that inhibition of caspases by overexpression of CrmA and Bcl-2, or by addition of the pharmacological inhibitor zVADfmk, prevented caspase activation and enhanced the recovery of cryopreserved cells (using DMSO). The caspase inhibitor was protective when added to both the freezing solution and the culture medium during the thawing process. Future application of hESC-based therapies would require similar strategies and protocols to reduce apoptosis and improve recovery of cells, through the use of biological CPAs like trehalose. Sasnoor et al.[53] reported prevention of apoptosis as a possible mechanism behind improved cryoprotection of hematopoietic cells.

## EFFECT OF CPAs ON EPIGENOMIC STATUS OF hESCs: DERIVATION, DIFFERENTIATION, AND PRESERVATION

Cryopreservation of human embryos has been a routine component of clinical *in vitro* fertilization (IVF) programs[54]. Frozen embryos are usually used in the treatment of infertility and the leftover lower-grade embryos are used in the derivation of hESC lines. However, there are no elaborate biological studies that address safety issues involving the cryopreservation of human embryos in toxic CPAs, and the use of such embryos in hESC derivation and translation to therapy. Epigenetic modifications in the cryopreserved embryos may lead to establishment of hESC lines with inherent genomic defects, many of which may remain undetected. This could also induce line-to-line variation across laboratories and inconsistency in the differentiation protocols developed by scientists. Applications of such cell lines in drug screening will also lead to ambiguous results with low reproducibility and may fail to mimic the events *in vivo*.

Recent advances in the study of physicochemical behavior of different cryoprotectants, use of various macromolecule additives in cryoprotective solutions and isolation, and use of proteins of plant and animal origin with antifreeze activity offer many new options for cryopreservation of oocytes and embryos of animal and human origin. Introduction of high dehydrating sucrose concentrations during cryopreservation of embryos was shown to increase survival and fertilization rates[55]. Rall and Fahy[15] obtained improved cryopreservation of mouse embryos by applying the vitrification method and by using sucrose as a CPA. In another study, Kasai et al.[56] followed the same method, but used trehalose instead of sucrose and obtained better results than sucrose. However, Eroglu et al.[57] used micromanipulation techniques to introduce trehalose inside the oocyte and then evaluated its effect on development of mouse zygotes. A quantitative microinjection technique was also developed using volumetric response of microdroplets suspended in dimethylpolysilaxene. At effective intracellular concentrations, trehalose did not impair development of mouse zygotes and it was suggested that use of trehalose in cryopreservation may give better survival rates[57]. However, to date, there is no conveniently adoptable protocol for safe permeabilization of trehalose inside the oocyte or embryo.

During embryogenesis, levels of DNA methylation are dynamically regulated by the *de novo* DNA methyltransferase Dnmt3a, Dnmt3b, and maintenance enzyme Dnmt1[58,59]. Failure to place or maintain the patterns of DNA methylation leads to early embryonic lethality in mice[60,61] and many human diseases including cancer, fragile-X, ICF, and ATRX syndromes (reviewed by Robertson[62]). In mouse ESCs, each of the highly expressed Dnmts plays a specific role in the establishment and/or the maintenance of DNA methylation[63]. Interestingly, mouse ESCs defective for DNA methylation can survive and proliferate in an undifferentiated state[64,65], but undergo rapid apoptotic cell death on *in vitro* differentiation[66].

A few reports establish the fact that the synthetic CPAs commonly used for preservation of ESCs and their derivatives can lead to epigenetic changes. It was found that addition of DMSO in culture (0.2–1.0%) to embryoid bodies (EBs) induced changes in the epigenetic profile, up-regulated Dnmt3a expression, altered genome-wide DNA methylation profile, and changed the phenotype of EBs[67]. Based on these observations, Iwatani et al.[67] proposed that the effects of DMSO on cells and on induction of lineages and fate should be interpreted by its effects on the epigenetic systems. During differentiation from ESCs, EBs establish a specific DNA methylation profile associated with both hypermethylation and hypomethylation at multiple loci[68]. Physiological and toxicological assessment of CPAs at the epigenetic level is, therefore, important, and analysis of genome-wide DNA methylation profiles before and after freezing hESCs will be useful in evaluating epimutagenesis.

## OFF-THE-SHELF STORAGE AND LONG-DISTANCE TRANSPORTATION OF CELLS AT AMBIENT TEMPERATURES

Storage of hESCs and their progenitors at room temperature, and their plausible transportation at ambient temperatures, would help to reduce the steps involved in cell processing (following freeze-thaw), and would improve cell survival and function. Such advances are also needed in order to address medical situations or emergencies that warrant immediate stem cell transplants. Cells stored and transported by air over long distances require large liquid nitrogen cylinders and are often surrounded by safety, security, and cost issues. In this context, trehalose has gathered considerable attention for its potential use as a highly efficient natural preservative (biopreservative). Some microorganisms are known to generate their own trehalose when they begin to desiccate. This protective capability of the sugar can also be induced in cells through artificial addition[25,34]. Even though water is required for the maintenance of biological integrity, numerous organisms are capable of surviving loss of virtually all their cellular water and existing in a state known as anhydrobiosis, and disaccharides (such as trehalose and sucrose) are largely involved in stabilizing these cells[69]. The ability to store cells in the desiccated state at ambient temperature would provide tremendous economic and practical advantages.

Gordon et al.[70] extensively studied the dehydration and storage of human mesenchymal stem cells (hMSCs). Three conditions were tested: air dried, air dried and stored under vacuum (vacuum only), and incubated with 50 m*M* trehalose + 3% glycerol and then air dried and stored under vacuum (vacuum + trehalose). Dehydrated hMSCs were shipped from San Diego to Baltimore overnight. On rehydration with normal hMSC medium, the cells were able to maintain high viability and proliferation capacity, and regain spindle-shaped morphology and adhesive capability. The cells were successfully passaged, tested for the expression of hMSC markers, and differentiated to different tissues. Although encouraging, these findings need to be evaluated for reproducibility and consistency, and tested for the duration of storage.

## TECHNICAL ADVANCES IN STEM CELL BANKING AND STORAGE USING TREHALOSE

Efficient cryopreservation is essential for widespread application of hESCs and for ES cell banking[6]. The creation of stem cell banks could help in the standardization of protocols for their culture and for conditions that could be tested in the cell lines deposited. hESC banks will help to standardize (1) measures of identity (short tandem repeat [STR] analysis, human leukocyte antigen [HLA] typing), (2) measures of stability (cytogenetic analysis, single nucleotide polymorphism [SNP] assays), (3) measures of pluripotency (gene expression profile), (4) epigenetic signature of cell lines, (5) toxicological screening, (6) cell line contamination, (7) cell line cross-contamination, and (8) proper database management. These banks will be able to distribute these protocols between the different research groups across the world. Well-preserved vials of hESCs may substantially elevate their cumulative applications in basic and therapeutic research. Effective freezing and storing of surplus cell vials also reduces the risk of shortage of cell lines.

Vitrification and controlled rate cooling methods were applied for the hESC cryopreservation and vitrification was found to be more effective than slow rate cooling[5]; however, slow rate cooling was more effective for mouse ESCs. This suggests that cryopreservation is cell type and species specific[71]. Two important procedures related to the use of permeating CPAs for the cryopreservation of cells are (1) the addition of a CPA to the cells before freezing and (2) the removal of the CPA from the cells after thawing. Cells transiently shrink when a CPA is added and then return to near-normal volume as the CPA permeates. They undergo transient volume expansion during the removal of the CPA, the magnitude of which depends on how the removal is effected and on the inherent permeability of the cell to water and CPA. In several cell types (e.g., oocytes), the addition of cryoprotectants (DMSO, or 1, 2-propanediol) has been shown to cause disruption of the cytoskeleton chemically[72,73]. Little is known about how intracellular structures and organelles respond to freezing, partly because it is difficult to assay their state and function *in situ* in a clear manner[74]. In a recent study, He et al.[75] showed that vitrification at a low concentration (2 *M*) of the intracellular cryoprotectant (1,2 propanediol) is a more effective approach than using 0.5 *M* extracellular trehalose for the cryopreservation of murine ESCs. In another study by Demirci and Montesano[76], mouse ESCs were encapsulated and vitrification was done at low cryoprotectant concentrations (1.5 *M* propanediol and 0.5 *M* trehalose). This method maintained high cell viability (>90%), and may have great impact on tissue engineering and high throughput screening, as well as therapeutics. However, similar studies in hESC cryopreservation are warranted.

Katkov et al.[77] reported that ESCs cryopreserved with DMSO show a down-regulation of the pluripotency markers, such as Oct-4, after thawing due to the mechanisms of stress-related events associated with cryopreservation. In an attempt to improve cryopreservation efficiency by modifying the conventional methods through addition of trehalose, Wu et al.[78] demonstrated improved recovery and normal expression of the regular hESC markers, after going through controlled rate freezing and rapid-thawing methods. The results indicated that the use of trehalose is efficient and convenient for cryopreservation of hESCs. Furthermore, Ji and coworkers[79] tried to reduce the time required to amplify frozen stocks of hESCs, minimize risk of clonal selection during freeze-thaw cycles, and facilitate storage of hESC clone libraries by loading adherent hESCs with the disaccharide trehalose prior to cryopreserving in a DMSO-containing cryoprotectant solution. This method increased cell viability by over an order of magnitude compared with cryopreservation in suspension and also reduced hESC differentiation.

### Use of Receptors and Transporters for Uptake of Trehalose

In another study by Buchanan et al.[80], trehalose was introduced into hematopoietic stem cells using the P2Z receptor, known to form nonselective pores in the presence of extracellular adenosine 5'-triphosphate ($ATP^{4-}$). Cells loaded with trehalose were then frozen, thawed, and evaluated for differentiation capacity and clonogenic output. Results obtained with this technique were compared to traditional freezing protocols using 10% (v/v) DMSO. Furthermore, there were no significant alterations in phenotypic markers of differentiation, activation, and proliferation. This report demonstrated that preservation of hematopoietic stem cell function with trehalose is superior to that obtained with DMSO, and this method could be widely adapted to any cell or tissue type expressing the P2Z receptor. Although it has not been investigated whether hESCs express P2Z receptors, we speculate that any molecule of the family would likely be up-regulated in hESCs owing to their role in cell death and immune regulation.

Similar attempts have been made by various groups for permeation of trehalose inside the cell membrane using different transporters. Several workers have used selected active sugar transporters *MalEFGK2* (TC: 3.A.1.1) and *MAL11/AGT1* (TC: 2.A.1.1.11) for bacterial and yeast origins that require either ATP hydrolysis or a favorable membrane potential for sugar transport[81,82,83,84]. However, these transporters have relatively broad substrate selectivity and include glucosides such as trehalose, sucrose, and maltose[81,82,85]. For both these transporters, the direction of transport of trehalose is only inward. The glucose transporter (GLUT)/SLC2A family of facilitated glucose transporters is known to permeate glucose across the cell membrane[86,87]. This transport is bidirectional between the external environment and cytosol, and is independent of pH and membrane potential[86,87]. However, these transporters are not trehalose specific. Kikawada et al.[85], therefore, isolated and characterized the functions of a facilitated trehalose-specific transporter (trehalose transporter 1 [TRET1]) from an anhydrobiotic insect, *P. vanderplanki*. *Tret1* cDNA encodes a 504-aa protein with 12 predicted transmembrane structures. *Tret1* expression was induced by either desiccation or salinity stress[83]. Expression was predominant in the fat body and occurred concomitantly with the accumulation of trehalose, indicating that TRET1 is involved in transporting trehalose synthesized in the fat body into the hemolymph. Functional expression of TRET1 in *Xenopus* oocytes showed that transport activity was stereochemically specific for trehalose, and independent of extracellular pH (between 4.0 and 9.0) and electrochemical membrane potential. These results indicate that TRET1 is a trehalose-specific facilitated transporter and that the direction of transport is reversible depending on the concentration gradient of trehalose. The extraordinarily high values for apparent *K*m (>100 m*M*) and *V*max (>500 pmol/min per oocyte) for trehalose both indicate that TRET1 is a high-capacity transporter of trehalose. Furthermore, TRET1 was found to work in mammalian cells, suggesting that it confers trehalose permeability on cells, including those of vertebrates as well as insects[85]. Cells and organs loaded with a high concentration of trehalose may be cryopreserved more efficiently and might even be lyophilized for preservation, such as in the case for human platelets[87,88]. All this evidence indicates that induced expression of TRET1 in hESCs may open many possibilities for successfully permeating trehalose inside the cells and improve their cryopreservation.

In another study by Buchanan and coworkers[89], a human hematopoietic cell line (TF-1) was used as a model for developing an improved method of human stem cell cryopreservation. Trehalose was loaded into the cells using a genetically engineered mutant of the pore-forming protein $\alpha$-hemolysin from *Staphylococcus aureus*, following a method described previously by Eroglu et al.[33]. This method results in a nonselective pore equipped with a metal-activated switch that is sensitive to extracellular zinc concentrations, thus permitting controlled loading of trehalose[89]. Preliminary experiments characterized the effects of poration on TF-1 cells and established optimal conditions for trehalose loading and cell survival. TF-1 cells were frozen at 1°C/min to –80°C with and without intra- and extracellular trehalose. Following storage at –80°C for 1 week, cells were thawed and evaluated for viability, differentiation capacity, and clonogenic activity in comparison to cells frozen with DMSO. Predictably, cells frozen without any protective agent did not survive freezing. Colony-forming units (CFU) generated from cells frozen with intra- and extracellular trehalose, however, were comparable in size, morphology, and number to those generated by cells frozen in DMSO. These data demonstrate that low concentrations of trehalose could protect hematopoietic progenitors from freezing injury and support the hypothesis that trehalose may be useful for freezing ESCs and other primitive stem cells for therapeutic and investigational use[89].

## Dry Desiccated Preservation with Trehalose

Recently, there has also been much interest in using trehalose and other small carbohydrates to preserve mammalian cells in a dried state as an alternative to cryopreservation. Studies on MSCs using Lucifer yellow (LYCH), a well-known probe used in the study of fluid-phase endocytosis, indicated the uptake process of trehalose and was inhibited at temperatures below 20°C. In addition, fluorescence microscopy revealed endosomes stained with LYCH accumulated in the MSCs and suggested that the dye entered the cytosol over time[31]. Trehalose was accumulated inside the MSCs, and its uptake was proportional to the length of incubation and showed a nonsaturating dependence on extracellular concentration. Endocytosis inhibitors were used to investigate the mechanism of uptake further. Colchicine and nocodazole, both of which depolymerize microtubules, blocked trehalose uptake. DMSO, which affects microtubules differently by forming shorter and more abundant microtubules, also inhibited trehalose uptake. However, cytochalasin B, which depolymerizes actin filaments and thus blocks both macropinocytosis and caveolae-dependent pinocytosis, did not cause a decrease in trehalose uptake. Amiloride, which blocks sodium channels and inhibits clathrin-independent pinocytosis, also did not inhibit trehalose uptake. Taken together, these findings suggest that hMSCs are capable of loading trehalose from the extracellular space by a clathrin-dependent fluid-phase endocytotic mechanism that is microtubule dependent, but actin independent[31]. Similar studies in hESCs may help to improve their preservation by devising alternative methods.

Chen et al.[90] reported on the successful preservation of plasma membrane integrity after drying as a first step toward full preservation of mammalian cells. Trehalose was introduced into cells using a genetically engineered version of α-hemolysin. The cells were then dried and stored for weeks at different temperatures with approximately 90% recovery of the intact plasma membrane. It was shown that protection of the plasma membrane by internal trehalose is dose dependent and the authors estimated the amount of internal trehalose required for adequate protection to be approximately $10^{10}$ molecules/cell. In addition, a minimal amount of water (approximately 15% wt) appears to be necessary. These results indicate that a key component of mammalian cells can be preserved in a dried state for weeks under mild conditions (–20°C and 5% relative humidity) and thereby suggest new approaches to preserving mammalian cells[91]. Furthermore, cryopreservation of cord blood cells under preclinical conditions was successfully carried out using trehalose as a supplement to DMSO[39]. The above reports indicate the disadvantages and toxic effects of commonly used CPAs like DMSO and glycerol, and suggest that trehalose may be a more suitable molecule for hESC cryopreservation, banking, storage, and transportation.

## Physical Methods for Intracellular Trehalose Uptake

Standard hESC cryopreservation methods, including slow freezing and vitrification, are plagued by poor viability and recovery of undifferentiated cells. Several alternative methods are therefore being tried. Mohr et al.[92] used electroporation as an efficient trehalose-loading and -transfection technique for cryopreservation of hESCs. Short pulse times of 0.05 msec facilitated trehalose loading; this loading efficiency per cell increased with multiple pulses. Ji et al.[93] developed a technique based on stabilizing the hESCs adherent to or embedded in matrigel matrix by loading them with trehalose prior to cryopreservation with DMSO. Very recently, Chakraborty et al.[94] reported a dry preservation method for mammalian cells using microwave processing. Cells preincubated with full complement media containing 50 m*M* trehalose for 18 h were dehydrated using intermittent microwave heating at 600 W, 30-sec intervals. Their study showed that intracellular trehalose provided protection against injury associated with moisture loss. The methodology also facilitates rapid and uniform dehydration of cell-based samples for dry preservations.

## SUMMARY

The use of trehalose in regenerative medicine remains unexplored. Besides being a natural cryoprotectant, trehalose can be used for the prevention and cure of neurodegenerative diseases. The use of trehalose along with embryonic or adult stem cells may enhance the recovery of patients suffering from such diseases. Stem cell cryobanking may be improved with the use of trehalose as a cryoprotectant and, at the same time, short-term storage of cells at ambient temperatures can be achieved by using trehalose. The ability to store cells at ambient temperatures for extended time periods will help in reducing the costs of developing stem cell–based therapies and would help in developing off-the-shelf storage of stem cells as drugs. With the recent FDA approval of hESC-based clinical trails, the need for developing such technologies for applications in hESC storage/preservation and transport is becoming increasingly vital. Such advances could radically change the science and business of tissue engineering and regenerative medicine.

## ACKNOWLEDGMENTS

Thanks are extended to Surjya Narayan Dash for the artwork. KDD thanks DBT, New Delhi, for support with the young scientist associateship in biotechnology. Internal funding from Manipal University, Manipal and Stempeutics Research Pvt. Ltd., Bangalore is also gratefully acknowledged.
